# Preparation of Cavities for Porcelain Inlays

**Published:** 1906-03

**Authors:** Geo. T. Banzet

**Affiliations:** Chicago.


### PREPARATION OF CAVITIES FOR PORCELAIN INLAYS.

By GEO. T. BANZET, B. S., D. D. S., Chicago.

The preparation of cavities for porcelain inlays differs materially from that necessary for gold or amalgam fillings, or gold inlays.

Absolute avoidance of distortion of the matrix upon removal from the cavity necessitates a formation in which the slightest undercut does not exist. Cavity preparation must be divided into various classes and each class considered separately.

The labial or buccal class consists of cavities involving the labial or buccal surfaces. The floor of these cavities should always be as nearly flat as possible, by which is meant that the floor at all points should be at almost right angles to the walls, and in some cases, at an absolute right angle.

The walls of the cavity should be as nearly parallel as will admit of the removal of the matrix without distortion.

Let us consider a particular cavity, say an upper central incisor, labial. The size of the cavity will of course depend upon the area of decay or erosion, but as most of these cavities are close to the cervix, the gingival margin should be almost invariably extended under the gum. The cavity should also be extended to the angles of the tooth, where the mesio-approximal and disto-approximal walls, being almost parallel, the floor of the cavity, following the curve of the tooth, will be at *right angles* to them.

It is advisable in many shallow cavities to make a slight groove at the junction of the walls and floor, which will stiffen the matrix, and increase the retention of the inlay.

The majority of approximal cavities of the six anterior teeth usually involve some other surfaces, as labial, lingual, or occlusal.

The preparation of these cavities should always, when possible, be from the lingual aspect. A proper separation having been secured, and remember that for the beginner it should be considerable, a matrix can be as easily burnished as where access is obtained labially, and the result will be more esthetic.

The cervical wall should be straight, the floor flat, and if the cavity extends over on to the lingual surface, the floor of the extension should be absolutely flat, and the end dovetailed, which will resist dislodgement from mastication.

A restoration involving the approximal, labial, and lingual surfaces should always be prepared from the lingual aspect, limiting the size of the inlay labially as much as possible. This is not only done for esthetic reasons, but also because frail labial walls can be retained with perfect safety and increased security to the inlay.

The immunity of inlays to recurrence of decay obviates the necessity of "extension for prevention," and it is unnecessary to extend the cavity as far cervically, or toward the angles of the tooth, as is necessary for a filling.

Do not try to secure absolutely straight margins, as irregular ones are less conspicuous. The cervical margins should be nearly straight, rather than semi-circular, and the junction with the axial margins should form an acute angle.

The margins should always be prepared at right angles to the tooth surface, which obviates thin edges of porcelain and prevents the cement from showing through.

Approximal-occlusal cavities of teeth posterior to the six anterior should be extended through the fissures of the bicuspids, and mesio-occlusal cavities of upper molars should be extended to the mesial fissures, and the disto-occlusal cavities to the distal fissures. Open the fissures to their extremity, as it makes a more permanent operation.

The axial walls of these cavities may be nearly parallel, the cervical wall and *floor* flat, the floor being depressed somewhat. Occlusally the cavity must be extended to the extremity of the fissure, or fissures and where necessary dovetailed. I wish to lay particular stress upon this point, as every approximo-occlusal cavity preparation should include the dovetailing occlusally, which will add materially to the retention of the inlay.

Porcelain may sometimes be used in occlusal cavities of lower molars, and the preparation is very similar to that of labial cavities, bearing in mind always that the floor must be flat and the walls nearly parallel.

In my own practice I never insert a porcelain inlay in a masticating surface where there is a liability of fracture of the margins.

If, for esthetic reasons, it be absolutely necessary to employ porcelain in such positions, it should be protected by reducing the occlusion.

There are many varieties of difficult compound cavities, which cannot be described in a paper of this extent, and which would be of no special value to beginners, therefore, I will not attempt them.

The subject of instrumentation is of importance in this connection, and will explain why some operators become more expert and expeditious than others. To secure the best results one must have instruments which are adapted to the work to be accomplished, and in this line of special work, special instruments are required.

Left hand burs, or burs made to cut when the engine is reversed, will be found of the utmost value. The most favorable shape is the inverted pear, and a variety of sizes and cuts should be procured.

The cross cut and plug finishing will be sufficient, and beautifully accurate preparations can be quickly secured by their use, in conjunction with the regular burs. One should also have a variety of sharp chisels with which cavity preparation can be very much simplified, and results secured in many instances in much less time than where the engine is employed. You should also have a number of very small gem, carborundum or corundum stones, and also some very small Arkansas stones for preparing and polishing the margins. Disks may also be employed in many positions for this purpose, but care must be taken to keep them at right angles to the tooth surface.

The margins cannot be *too good* as the more carefully they are finished the easier will it be to burnish the matrix, and the more perfect the adaptation of the finished inlay.

In conclusion I wish to impress upon your minds the "rule of three," keep the floor flat, make the walls as nearly parallel as will admit of the removal of the matrix without distortion, and finish the margins at right angles to the tooth surface.

